# Exploring cross-boundary collaboration for youth mental health in Sweden – a qualitative study using the integrative framework for collaborative governance

**DOI:** 10.1186/s12913-024-10757-y

**Published:** 2024-03-11

**Authors:** Linda Richter Sundberg, Anne Gotfredsen, Monica Christianson, Maria Wiklund, Anna-Karin Hurtig, Isabel Goicolea

**Affiliations:** 1 Department of Epidemiology and Global Health, Umeå University, Umeå, Sweden; 2 Department of Nursing, Sexual and Reproductive Health, Umeå University, Umeå, Sweden; 3 Department of Community Medicine and Rehabilitation, Section of Physiotherapy, Umeå University, Umeå, Sweden

**Keywords:** Youth mental health, Youth mental health services, Mental health system, Collaboratio, Governance

## Abstract

**Background:**

Youth mental health is a major health concern in almost every country. Mental health accounts for about 13% of the global burden of disease in the 10-to-19-year age group. Still there are significant gaps between the mental health needs of young people and the quality and accessibility of available services. Collaboration between health and social service actors is a recognized way of reducing gaps in quality and access. Yet there is little scientific evidence on how these collaborations are applied, or on the challenges of cross-boundary collaboration in the youth mental health space. This study aims to explore how collaboration is understood and practiced by professionals working in the Swedish youth mental health system.

**Methods:**

We conducted 42 interviews (November 2020 to March 2022) with health and social care professionals and managers in the youth mental health system in Sweden. Interviews explored participants’ experience and understanding of the purpose, realization, and challenges of collaboration. Data were analysed under an emergent study design using reflexive thematic analysis.

**Results:**

The analysis produced three themes. The first shows that collaboration is considered as essential and important, and that it serves diverse purposes and holds multiple meanings in relation to professionals’ roles and responsibilities. The second addresses the different layers of collaboration, in relation to activities, relationships, and target levels, and the third captures the challenges and criticisms in collaborating across the youth mental health landscape, but also in growing possibilities for future development.

**Conclusion:**

We conclude that collaboration serves multiple purposes and takes many shapes in the Swedish youth mental health system. Despite the many challenges, participants saw potential in further building collaboration. Interestingly our participants also raised concerns about too much collaboration. There was scepticism about collaboration directing attention away from young people to the professionals, thereby risking the trust and confidentiality of their young clients. Collaboration is not a panacea and will not compensate for an under-resourced youth mental health system.

**Supplementary Information:**

The online version contains supplementary material available at 10.1186/s12913-024-10757-y.

## Background

Youth mental health has emerged as a major global public health concern during the last two decades [1, 2]. Amplifying the already significant challenges in youth mental health, Covid-19 has impacted heavily on the mental health of adolescents and young adults [3, 4].

Research shows that the youth mental health system is incompatible with young people’s needs. Studies highlight significant gaps between the needs for preventive and curative mental health care and available accessible services [5, 6]. Low access to youth mental health services is associated with multiple barriers at societal and system levels [7]. In addition, trust, mental health literacy and social stigma, systemic structural barriers such as high costs and staff turnover, are obstacles to young people’s access to mental health services [8, 9]. Moreover, the increasing differentiation and specialization of contemporary youth mental health services has worsened fragmentation, consequently impacting on service access [10].

In light of the above, efforts to transform and improve the youth mental health system have included an increased focus on the coordination of services and collaboration within and between sectors. As evidenced in research [11] policies and practices [12, 13] *collaboration* has emerged as a key strategy for improving the access and quality of youth mental health services in the last decades.

Policy-makers and researchers have high expectations regarding the benefits of collaboration [14, 15]. The collaborative concept is central to many current strategies aimed at improving the quality and accessibility of youth mental health services. Yet scientific evidence on the meaning and actual doings of collaboration from the perspective of those working within youth mental health, is lacking [16].

### Cross-boundary collaboration in youth mental health care– theory and concepts

For a concept so widely used in everyday language there is a surprising lack of a clear understanding of what it is to collaborate, and of how best to support and improve collaborative working. Definitions are often tailored to a particular environment. [16, p.e1]

Definitions of collaboration have been debated and several scholars have attempted to clarify the concept. Collaboration has been defined as an “integration of activities and knowledge that requires a partnership of shared authority and responsibility” [14, p.208].

Collaboration in the context of health and social care comprises sub-concepts which depend upon context and type, e.g., interprofessional collaboration, and interagency collaboration [17–19]. Interagency collaboration in the context of child and youth mental health refers to “the process of agencies joining together for the purpose of interdependent problem solving which focuses on improving services to children and families—represents a fundamental reform in the way services are provided for children with serious emotional disturbance and their families” [20, p.292]. Interagency collaboration can take place on multiple levels, from the frontline among health professionals, social workers, families, teachers, and others, to relationships between policy-makers and administrators responsible for addressing organizational mandates, financing, and management. Interagency collaboration can involve public, private, and/or non-governmental entities as partners [21] and is closely related to the concept intersectoral collaboration which refers to collaboration between sectors such as health care sector, educational sector or social services [22].

Sullivan’s *collaborative practice* [23] highlights the four elements needed for good collaboration in health care: (i) coordination towards shared goals; (ii) cooperation that acknowledges and values the contribution of others; (iii) shared decision-making relying on balanced negotiation, trust, and respect, and (iv) partnerships that are open and respectful and develop over time.

Collaborative care models typically aim to facilitate and formalize interorganizational or intersectoral collaboration by offering guidance on which services and activities to include in collaborative care processes. One example is interprofessional collaborative practice, which implies an explicit partnership between health care providers, patients, and their families in coordinated collaborative activities with shared decision-making [24].

An inter-related concept is that of boundary spanning, which describes how actors work beyond their own structural social and cultural groups to deliver public information and services [25, 26]. They act as both connectors and gate keepers, with the capability of establishing, transgressing, maintaining, negotiating, and dissolving boundaries between different organizations [27, 28].

During the first decade of the millennium many countries formalized collaboration in the realm of mental health and social care through legislation and explicit collaborative models, such as the community youth mental health hubs [29–31]. Collaboration and collaborative care models are associated with expectations of increased synergies, and improved coordination and quality of service provision [32–34]. Collaboration has also become a means and an indicator of quality in many health care systems [35].

### The integrative framework for collaborative governance

Governance is a collective action with joint formal or informal agreed norms to guide individual and group behaviors [36]. Emerson et al., define collaborative governance as “the processes and structures of public policy decision making and management that engage people constructively across the boundaries of public agencies, levels of government and/or the public, private and civic spheres in order to carry out a public purpose that could not otherwise be accomplished.” [37, p.e2].

**Fig. 1:**
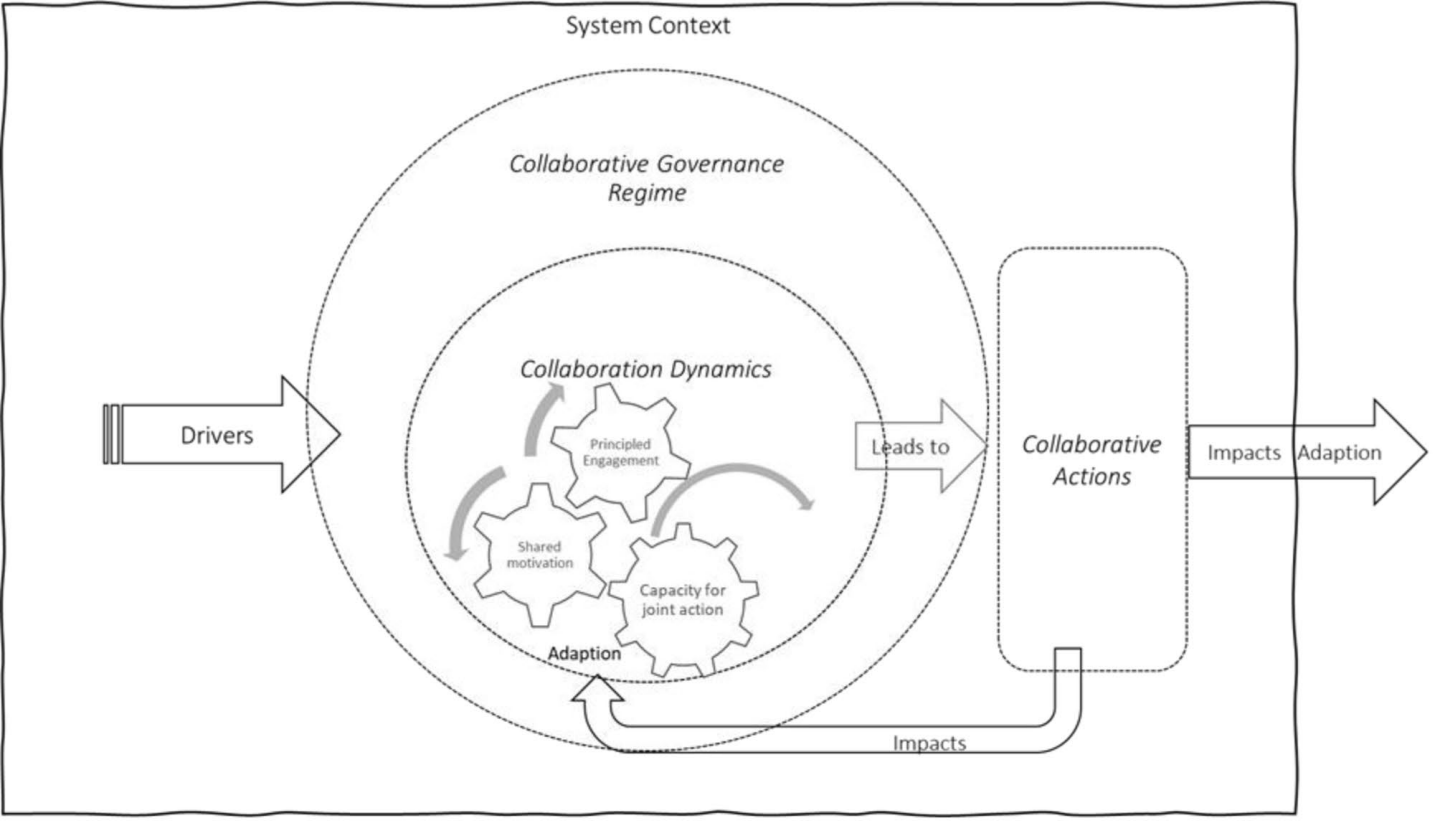
The integrative framework for collaborative governance, adopted from [37]

Emerson’s Integrative Framework for Collaborative Governance (IFCG) comprises three interwoven dimensions *– Collaborative Governance Regime, collaborative dynamics, and collaborative actions*. See Fig. 1.

The *collaborative governance regime* refers to the system of public principles and decision-making in which boundary-crossing, collaborative activities and behaviors take place. The other two dimensions– *collaborative dynamics* and *collaborative actions -* are within the collaborative governance regime. Collaborative dynamics entails the three components - principled engagement, shared motivation, and capacity for joint action. They work together to generate collaborative actions to realize the intention of the collaborative governance regime. The separate dimensions and components of the IFCG are depicted with dotted lines that illustrate the interactive and reciprocal relationships [37].

The IFCG also includes a surrounding system context indicating that legal, political, socioeconomic, and other aspects, through drivers such as leadership, incentives, and uncertainties, create constraints and possibilities for collaboration in both short and long term time frames. The system context not only influences but is shaped by the collaborative dimensions [37].

### Aims and research questions

The issue of youth mental health is gaining increasing attention in research and policy-making. Although collaboration is promoted across health and social services, research exploring collaboration within the youth mental health space is scarce. The current study addresses this knowledge gap by exploring how collaboration is understood and practiced by professionals working in the Swedish youth mental health system.

The following questions are explored:

1. What is the role of collaboration in the youth mental health system from the perspective of youth health professionals?
2. How is collaboration practiced in the youth mental health system?
3. What challenges and possibilities are linked to collaboration in youth mental health?

## Methods

### Study design

Data were analysed under an emergent study design using reflexive thematic analysis [38, 39]. Forty-two interviews were conducted with health professionals working in different parts of the Swedish youth mental health system.

### Study setting and participants

**Fig. 2:**
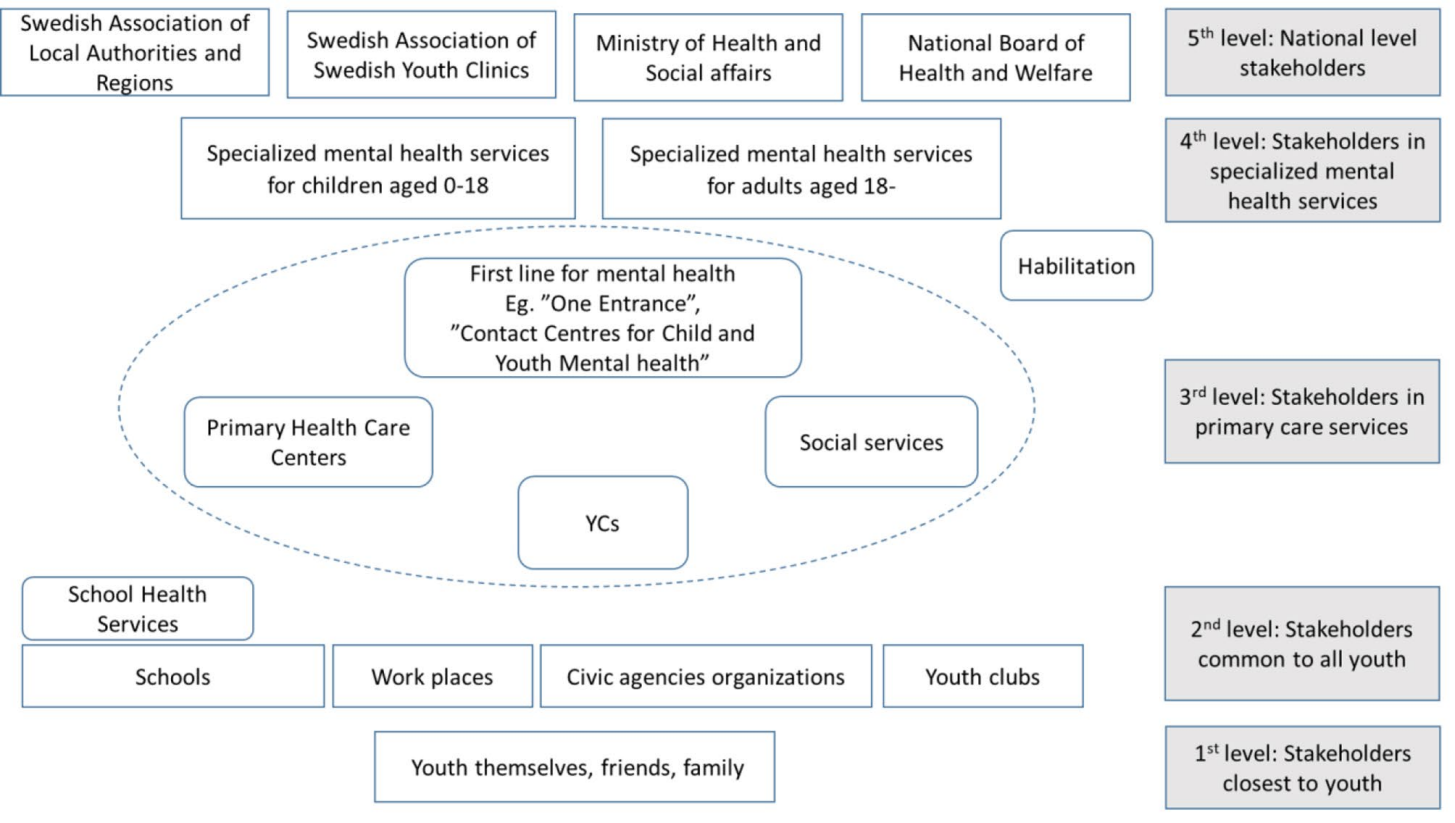
The youth mental health system in Sweden

In Sweden, as in many other western countries, the youth mental health system operates as an integrated part of the larger health system that is governed by national government. Health care is provided by primary and specialized health care organized in 21 regions. For more information about the governance of the Swedish youth mental health system, see appendix 1 The youth mental health system in Sweden (Fig. 2) targets adolescents and young adults in the age span 13–26 years old. Figure 2 depicts young people and their social networks of family and friends at the first level. Organizations that target the entire youth population, e.g., schools, workplaces, leisure associations, school health services, youth clubs and non-profit organizations, are shown at the second level. ‘First line’ primary health care, which includes health centers, youth clinics (YCs) and social services, is at the third level. Specific clinics for young people’s mental health have been established, or are about to be established, in many parts of the country. They include ‘One Way In’, ‘Children and Youth 7–17’ or health centers assigned to address youth mental health. Specialized health care, such as child and adolescent psychiatry (up to eight years) and adult psychiatry (18 + years) is depicted at the fourth level. The fifth level includes the national actors i.e., the Ministry of Health and Social affairs, the National Board of Health and Welfare, the Swedish Public Health Agency, the Swedish Association of Local Authorities and Regions and the Association for Swedish Youth Clinics.

The levels and borders are not absolute because the actors and their responsibilities differ and change across levels and services [40].

Health services on all levels are expected to collaborate to offer the best possible care for young people. However, collaboration in the Swedish context has encountered many challenges [41].

The interviews were conducted in three of the 21 regions in Sweden. These regions shared many characteristics due to being in the same national health care system, but they also differed. For more detailed information about the three regions, see supplementary file 1. This study is part of a larger research project analysing the role of YCs in youth mental health care in Sweden [42].

### Data collection

The three regions were selected using a purposive sampling strategy which aimed to identify and recruit participants who could provide rich and diverse data to address the research questions. Our intention was to include health care regions offering variation in size and geographical location. The researchers approached a larger region, a smaller region and a medium sized region located in the northern, southern, and middle part of Sweden. Organizations operating in the regional youth health system were approached within each region. Professionals with experience working in youth mental health were invited to take part in the interviews through the managers of the organization. The organizations themselves nominated participants that they assessed working with youth mental health. Data were collected from November 2020 to March 2022. A total of 42 semi-structured interviews were conducted with health and social care professionals and managers working in the Swedish youth mental health system. See Table 1.

**Table 1:** Informants - Regions and professional groups

	Region 1	Region 2	Region 3	Total
Professional group, organization	Midwifes, YC (2) Physician, YC (1) Counsellor/social worker, YC (3) Local and District Manager, YC (1) Psychologist Child and Adolescent Psychiatry (1) Psychiatrist, Adult Psychiatry (1) Social Worker, Social Services (1) Counsellor, School health (2) Nurse, School Health (1) Psychologist, School Health (1) Manager, Youth Theatre (1) Social Worker, youth café (1)	Psychologists, YC (2) Counsellor/Social Worker, YC (3) Nurse/sexologist, YC (1) Nurse, YC (1) Local Manager, YC (1) District Manager, YC (1) Manager and Psychologist at Youth Psychotherapy Center (1) Local Manager, Leisure Organization (1) Local Manager, School Health (1) Counsellor and Midwife– Outreach YC (2) Psychologist, Gender Agency (1) Psychologist, Trauma Agency (1) Psychiatrist, Child- and Adolescent Psychiatry (1)	Counsellor, YCs (2) Manager responsible for YCs in the Region (1) Manager Medical Leadership, YC (1) Manager Psychosocial Leadership, YC (1) Manager School Health (1) Manager of Child- and Adolescent Psychiatry (1) Manager of Leisure Organizations for Youth (2)	
Number of participants	16	*17*	*9*	42

Four researchers (LRS, AG, MC, IG) with experience in qualitative interviewing conducted the interviews in Swedish, either via Zoom or telephone. Face-to-face interviews were not possible due to the Covid pandemic. The approximate length of the interviews was one hour. A semi-structured interview guide was used to elicit participants’ views and experiences of interorganizational collaboration in the context of youth mental health. See example of interview guide in supplementary file 2. The data collected for this study has not been published elsewhere.

### Data analysis

The transcribed interview material was entered into software used for qualitative analysis (NVivo 10) and analyzed following a modified version of Braun and Clarke’s six phases for conducting thematic analysis [37]. The modification implied that the sixth step– ‘producing the report’ was integrated with the steps 3–5 (i.e., searching, reviewing, defining, and naming the themes).

In the first phase of the analysis, we familiarized with the data by reading through the transcribed interviews several times and making preliminary notes. Initial ideas were then discussed by the research team (LRS, AG, MC, MW, AKH, IG). In the second phase, the first and last author (LRS & IG) divided the interview transcripts and read them again in more detail. During this phase we also started to systematically code features of the data that were related to the research questions. The initial codes were first discussed between LRS & IG and then within the larger team (LRS, AG, MC, MW, AKH, IG). These discussions guided the third step in which codes were organized into preliminary candidate themes. At this stage the IFCG [36] was used to further analyze the collaboration and understand manifestations, challenges, and possibilities in the Swedish youth mental health system. The IFCG fitted well as it offered opportunities to analyze the boundary spanning collaborative processes across different types of organizations. Parts of the text for this manuscript were also drafted at this stage.

In the fourth phase we moved back to the codes and interviews to check whether the candidate themes captured all the material. Some revisions were made, and a thematic map of the analysis was developed. Revised candidate themes, including a thematic map, were discussed with the research team. This led to the fifth step, which involved naming and defining each theme. Again, we went back to the initial codes and transcribed interviews to ensure that the thematic structure was grounded in the data. We also identified illustrative authentic quotes to help readers to understand the results. During this phase we discussed additional theoretical concepts to underpin and broaden our understanding of the findings. The team provided comments on the manuscript drafts. The final version was approved by all members of the team.

### Ethics approval and consent

Measures were taken to protect the rights and integrity of the participants during all phases of the study. All methods were carried out in accordance with relevant guidelines and regulations and the Declaration of Helsinki. Written informed consent was sought and gained prior to each interview. Ethical approval was granted by the Swedish Ethical Review Board (Dnr 2019–02910, 2020–04720).

## Results

The analysis produced three themes. The first describes how collaboration in youth mental health care is considered essential and important, and how it serves diverse purposes and holds multiple meanings in relation to professionals’ roles and responsibilities. The second theme addresses the different layers of collaboration in relation to activities, relationships, and target levels. Finally, the third theme addresses the challenges and criticisms of collaborating within the youth mental health landscape, but also the possibilities for further development.

Collaboration as crucial and serving multiple purposes

Collaboration is important, both internally here within social services, but also with other organizations. We collaborate with specialized mental health services, primary health care, schools, and many other actors. I think for mental health, collaboration is crucial, especially as the system is poorly equipped and under resourced to deal with this problem. No-one can do everything, but everyone can do something.

Social Worker, Social Services, Region 1

This theme focuses on the purposes and meanings that the participants attributed to collaboration in the youth mental health landscape. Collaboration in this context connects multiple actors in networking patterns, allowing them to join efforts in working towards common goals. As the above quote exemplifies, collaboration was depicted as crucial, and participants provided several diverse arguments as to why collaboration was particularly important within the youth mental health space.

Because sometimes when it comes to mental health problems, a referral needs to go straight away because you notice that this is too complex or other agencies are needed that can help the youth in another way. We also refer and try to use many different instances, such as support centers within the municipality. There are other organizations that target family abuse, eating disorder units etc.

Psychologist, YC, Region 2

The centrality of collaboration was connected to participants’ views of the concept of youth mental health and the system in which it operates. They see youth mental health as a *holistic problem or phenomenon*, that calls for multiple perspectives, multi-sector involvement and multi-actor efforts. This holistic perspective also implies that a variety of youth health (and social) needs must be addressed, including, for example, social, economic, psychological, physical, and sexual aspects. The centrality of collaboration in the youth mental health system is also linked to the narrative around mental (ill) health as an *intrinsically complex issue* that requires the engagement of multiple perspectives and disciplines to be properly understood.

Participants saw that collaboration had several purposes. The first involved helping young people, parents, and staff to navigate between what are sometimes detached services, clarifying the different roles that each actor can play in the system, and smoothing referrals for seamless transitions across services.

Collaboration is very important, and I can imagine that as a young person or parent, you may be in a difficult situation when you need help from different places and it can be incomprehensible, all these boundaries and so on. If the services who are involved around a family, can work together, I think that facilitates enormously. So that the current situation and the planned services from society become understandable. I can meet families sometimes where they barely know where they have been. In the end, they say BUP (Child- and adolescent psychiatry) about everything, because they cannot keep track of everyone. They do not know where they have been.

Psychologist, School Health, Region 1

Smoothing referrals and transitions were seen as being especially relevant for collaborators from organizations outside the health care system. While specialized services for children, adolescents, and adults and, to a certain extent primary health care and YCs, have mental health care as their core assignment, most of the services and organizations attended by young people (e.g., recreational spaces, leisure organizations, and schools) do not. Collaboration was seen as the core way of enabling these other bodies to adequately refer young people to appropriate services to meet their mental health needs.

Secondly, collaboration also served as ‘arena’ for negotiating and sharing responsibilities and knowledge between different youth mental health services. Collaboration allowed opportunities to share responsibilities and tasks. This benefitted overloaded services.

Third, collaboration was also perceived as a way of reaching specific groups of young people that might otherwise not have been reached by the health system. One such example is how one of the regions had analysed which groups of young people had accessed services less often and may therefore have unmet needs. This strategy then became the basis for collaboration with different organizations and institutions to reach out to marginal groups in need of services.

We know from surveys that we have difficulties in reaching youth who identify as boys and young people with trans experience. Further, we don’t reach youth with function variations and youth living in socio-economic vulnerability, newly arrived or newly Swedish young people. So, we prioritize our collaborative efforts to target these groups of youth. We go out to the high schools and give one and a half hour sessions where we talk about mental illness and sexual health and sexual consent. And we try to prioritize schools where we know that there are many immigrants and high schools where many students identify as boys or and the language introduction programs.

Nurse, YC, Region 2

Finally, collaboration was also used as a strategy to illustrate and compensate for the under-resourced youth mental health system. Collaboration aimed to compensate for gaps between specialized and community-based services or in the transitions from child to adolescent and adult services. Participants described how collaboration also served to make the gaps and inadequacies in the system more visible and obvious.

### Collaboration is multi-layered in the youth mental health system

This theme focuses on how collaboration occurred within and across different layers within the youth mental health system. The analysis identified three layers: *the layer of activity*, *the layer of relationship*, and *the layer of target levels.* These layers do not operate separately but interact and overlap. For example, how collaborative relationships were initiated and established varied depending on other layers of collaboration, such as the type of activity being developed.

#### The layer of activity

Collaboration in youth mental health is often described as doing something, an *activity, or a series of activities*. While participants gave examples of collaboration for prevention and promotion *activities* (for example working with schools) the focus was on how to collaborate with and *respond to* mental health problems in a timely manner to enable this to occur as early as possible.

It is often a matter of working together in these cases. For example, in joint follow-up meetings. What do you do and what can we do? And how do we do this? And then you follow it up after a while and so on. When we can do it like that, it often gets good, I think. That we do it together, and that we are sort of clear with who does what and so on.

Social Worker, Social Services, Region 1

As depicted in the above quote, in many cases, collaborative activities manifested in preparations for participation in meetings with multiple professional actors from the youth mental health system. Collaborative activities included what participants described as “working together” which could imply, for example, sharing information about the young person and their needs and situation. Working together also implied a sharing of responsibilities, coordination of different interventions and planning upcoming meetings.

Collaboration was in many cases centered around specific referrals to specialized services. However, there were complaints from staff at the primary or community level that their referrals did not always reach the intended service, and they did not know how to counter this.

To send a referral to another place is not so much a collaboration really, it’s just that you send a referral. But when you get referrals back and you do not understand why, that’s when you need a collaboration, because then you need to be able to communicate. It’s good to know the status in terms of waiting times or what referrals should look like. This is about exchanging experiences, how do we work, how do you work? We had that before, but not anymore.

Physician, YC, Region 1

As the above quotes show, collaboration included the *activity* of defining or clarifying the professional role or the role of the organization that collaborators represented. This part of the collaborative process could sometimes lead to negotiations and discussions on collaborators’ responsibilities in relation to one another, an aspect that leads to the next layer - that of r*elationships*.

#### The layer of relationships

Collaboration was also about building and engaging in *relationships* between one or several actors with a common goal, task or question in relation to youth mental health. The relationships were in some instances formed by explicit, formal and lasting agreements, other times the agreements were implicit and informal.

Collaborative relationships were perceived to be in different combinations, with various possibilities and limitations. Less formal collaborations were easier, more emergent and temporary in character, often growing from needs driven by the young people. One example of less formal collaboration was when professionals in YCs who had prior experience in specialized services, sought contacts from their previous work, to help ‘smooth’ collaboration for individual cases.

Now it was much easier for me because now I have these contacts since before. Then I could call and say ‘You know what, now I’m going to send a referral, go in and check all the records, I don’t really know what we’re going to do here but we have to talk about it and agree on something ’we can’t pass this youth back and forth anymore’ […] it can save us time when you get these personal contacts between different instances.

Counsellor YC, Region 3.

Some of the formal collaborations cited emerged from institutionalized or formalized agreements and were linked to organizational or legal structures, e.g., Coordinated Individual Planning (CIP) meetings that are arranged when an individual has need of support from several social and/or health sectors. Invited participants have legal obligations to participate in CIP-meetings. Team conferences are further examples.

Nowadays we collaborate mainly through CIPs. Someone initiates a CIP contact and then we are legally obliged to follow them. I would like to say that all the collaboration that we are part of is initiated through CIP.

Psychologist, Child and Adolescent Psychiatry, Region 1.

Regardless of whether they are formal or informal, collaborative relationships can be either ‘shallow’ or ‘dense’. Shallow collaborations implied that actors merely knew about each other’s existence or that collaboration was limited to short referrals or brief telephone contacts. In contrast, dense collaborations were characterized by several, extensive, joint activities. Here collaborations are more long-lasting (e.g., the YC and school health arranging annual anti-stress education). It was also stated that these denser collaborative relationships depended on authentic personal contacts or relationships that extended beyond formalized structures and compulsory collaborations.

#### The layer of target levels

A further layer of collaboration was the *target* level, meaning that collaboration was described as (potentially) taking place at individual, professional, managerial and/or system levels.

A substantial part of the collaborations described by the participants concerned the individual. This type of collaboration could be initiated by any of the actors in the youth mental health system; the collaboration can take place on one occasion or extend over several years. An example of individual level collaboration is CIP meetings. Here the focus is on one young person; different professionals discuss and coordinate the services provided by different actors to meet the needs of the individual.

Closely related to this type of collaboration was collaboration at the level of professionals. This included activities where professionals working in different services engage beyond individual cases. One example is meetings between social workers and school health staff, where more general health issues were addressed, e.g., school absenteeism and drug use. Another was the coordinated work between YCs and schools in organizing promotional visits to YCs. The focus was on learning, smoothing referrals, joint activities and knowing one another’s work. One example of this was a collaborative group of counsellors focusing on the prioritized development of youth mental health issues.

We have a local working group, that develops and implements care programs for depression and anxiety. And then there is an investigative working group that is composed of different people from all over the region with different skills and in different activities to look at difficult issues that usually fall between the chairs. And then we get assignments from a steering group that is county-wide and that is both municipal and regional activities.

Manager Psychosocial YC, Region 3.

The next target level was at the managerial or system level. Here it is important to note that in certain services, managers are also professional service providers blurring the distinction between professional and managerial staff. The focus of this collaboration goes beyond clinical and daily work in setting agreements between services that help define the collaboration - why, when, with whom etc.

Collaboration on individual, professional, managerial, and system levels interacts. Individual and professional level collaborations are (sometimes) nested in the larger organization or system (managerial and system collaborations). As mentioned above, collaboration on individual or professional levels can depend upon agreements at organizational levels. In response, the organizational level collaboration is, in some instances, developed from the needs of individuals or groups of young people. There are also descriptions of how individual level collaboration ‘tests’ how organizational level collaboration functions. Collaborations concerning groups of young people in other spaces often derive from the observed needs of several individuals.

### Many gaps and a lot of potential– the challenges and opportunities of collaboration on youth mental health

This theme outlines how participants experienced the challenges of collaboration and the consequences of weak collaborative processes. It presents participants’ experiences of good collaborations and their ideas on the development of collaboration within the youth mental health system.

While collaboration was perceived as being essential, participants specifically described challenges in relation to *actual* collaboration. These challenges were depicted in three inter-connected areas: (i) diverse views and perspectives on youth mental health among collaborative actors, (ii) lack of agreements, and (iii) an under-resourced mental health system that struggles to prioritize collaboration.

#### Diverse views and perspectives on youth mental health complicates collaboration

Participants described how the different roles of each organization also somehow implied different views on youth mental health and the types of support that was needed.

We are so clearly working on the youth’s mission, we do not want to be invited to a meeting that the young person has not chosen that we should be involved in. In one example, the social services had a case with a young person. They wanted to invite the YC to a meeting where we would talk and present and say that ‘you now can come to us and talk about these things’. And we did not want to do that in that context, with social workers, staff from school etc. No, absolutely not.

YC Manager, Region 3.

In the above quote above the manager of all YCs in Region 3 explains the clash between the YC approach centering on young people, and approaches taken by other services which she perceives as paternalistic. The YC way of collaborating could sideline young people by placing the focus on dialogue and collaboration *between professionals.* This approach was seen as not acceptable.

During the interviews participants described challenges related to the fact that collaborative actors often held different views on youth mental health. These different views sometimes led to diverse perspectives on both the goals and means of collaboration to the extent that this actually hindered collaboration. For example, YCs and school health services commonly expressed a non-medical view on youth mental health, one which focused on preventive and salutogenic factors that are concerned with well-being rather than disease. This view was perceived as clashing with the rest of the health care system (primary health care, specialized mental health services) that had a medical disease-orientated perspective. The YC approach to young people as autonomous and independent and their hesitancy to engage parents and/or guardians, was also perceived as being ‘at odds’ with the involvement of guardians/parents in specialized mental health care.

Some saw that different views on central concepts and aspects introduced friction to collaboration. On the converse, sharing similar perspectives (as for example the salutogenic focus and the approach to young people as autonomous individuals shared by schools and YCs) often led to long and productive collaborations.

#### The lack of agreement at the managerial level

Participants described how the lack of agreement between higher level managers representing the different collaborative actors, challenged several aspects of the collaboration. The function of these agreements was to justify or approve collaboration and to clarify roles and responsibilities. It also served the purpose of relieving the involved youth (and sometimes families) the burden of listening to professionals and negotiating responsibilities and finances.

Because you don’t want to sit on CIPs where you juggle responsibilities or money with each other. And that youth needs to listen to that. The overarching issues on economics and responsibilities should be settled on higher levels in the organizations. So that the therapists and the social workers do not end up in those discussions.

Local Manager, Social Services, Region 1.

Further, as many of the collaborative organizations were operating with heavy workloads, the systemic agreements implied that the managers gave clear signals that collaboration should be a priority and not an optional activity.

Without these higher-level agreements collaboration became something that was up to each individual professional to arrange, making the impact of personal contacts (informal collaboration) more prominent.

Sometimes it happens that we or I arrange collaboration meetings anyway. Because I think it’s so incredibly important. But I can only do that when I have the extra time and then I involve the people I already know from my previous workplace.

Counsellor, School Health, Region 2.

As the counsellor in the above quote describes, personal commitment can compensate for the lack of overall agreements, but such compensations are unsustainable in the long-run.

#### An under-resourced youth mental health system challenges collaboration

In many cases, the reason for poor collaboration adds to the difficulties in several of the collaborative organizations.

I believe very much in those collaboration meetings. And they are the most difficult to achieve in a way because we have so much to do. Everyone has a lot to do. Youth and adolescent psychiatry have a lot to do. The health centres…. The youth clinics have a lot to do.

Coordinator, School Health, Region 2.

Due to heavy workload health professionals prioritized the core mission of their own organization and were unable to see how they could prioritize collaboration. This has a ‘spillover’ effect to other institutions, e.g., if Child and Adolescent Psychiatry does not come to the collaboration meetings, the other actors start wondering whether they should be also skipping those meetings. At the same time when institutions do not engage in collaboration and work in isolation, this also contributes to making their workload heavier, which perpetuates a vicious cycle.

Participants also described how sometimes each organization guards its borders and sits in its corner.

I think it’s a kind of mistrust between us. You think that others should do this or that. And that there is perhaps a lack of clarity as well, whose responsibility it really is. It can be, for example, youth with anorexia, which is a serious illness. We might think that is the responsibility of psychiatry, but they might say it’s a lack of parenting skills. But maybe we have to do it together. We have to see the whole package. Let’s help them.

Social Worker, Social Care, Region 1.

Collaboration is then hampered by the strategy to push the responsibility towards others which can eventually become the basis for mistrust.

#### When does it work?– experiences and ideas on a strengthened collaboration for youth mental health

Participants also described well-functioning collaboration or collaboration processes that have been improved. Well-functioning collaborations were characterized by the presence of overarching agreements, professionals who know the cases and were committed, and actors engaging in collaboration across organizational borders.

If I try to think about a good collaboration meeting around an individual youth. Then there are people sitting there who have the mandate to be able to make decisions and who can do it at seated tables and are prepared to do so. […]. And bad collaboration, that’s when it’s people who don’t have the mandate to make decisions, who say ‘no, but I have to take this back to my organization’ or ‘I must check with my boss’ or who are not well-prepared, who do not know the family but have only stepped in and are at a meeting just to sit out. There is poor collaboration.

School Health Manager, Region 3.

When collaboration is part of, or the result of, overarching decisions and agreements, participants perceived that it worked better. Collaborative agreements have the function of identifying actors, issuing mandates and also integrating the collaboration within the participating organization. Through such agreements collaboration becomes a part of the mission and role of each organization. Participants provided several examples of how this led to improved collaboration and less problems with low attendance or collaborators refraining from collaborative activities.

We have a formal collaboration agreement in writing between the schools and child- and adolescent psychiatry. This document is important. The low adherence from psychiatry in our meetings changed after this agreement was formed. All actors can refer to the document where the division of responsibilities is formulated, we can evaluate how the collaboration works and should be developed.

Counsellor, School Health, Region 1.

Other characteristics of collaboration that work well include trust, knowledge about what each service does, hands-on practices (concrete collaboration, not only overarching agreements) and personal closeness between collaborators, in contrast to person-bound collaboration that depends on specific people. Having enough time and resources for each service so that collaboration does not have to compensate for gaps, having actors appreciate each other’s roles, feeling appreciated and understanding and taking responsibility for their roles in the network, are all important factors. Another strategy that facilitated collaboration was when organizations had explicit ‘entry’ into collaborations, e.g., a designated telephone line or a web-based contact center for professionals.

Thus, through collaboration participants engage in a process of discovering and defining a common problem to be solved, and how the complexity of the problem justifies collaboration as the adequate way of addressing it. Despite the consensus that the more collaboration the better, there were a few critical voices.

But it is also very easy to say. ‘Yes, but it is collaboration that is lacking. If only people wanted to cooperate better ‘. There are ‘drainpipes’ in municipalities and health centres and everywhere. So yes, that’s true in a way, of course. But in another way, it’s also easy to put everything there. So, then it will be… Yes, care would be more effective if everyone collaborated better.

Manager Youth Psychotherapy Center, Region 2.

Collaboration, as the manager expressed in the above quote, can battle the “drainpipes” of youth mental health services, and contribute to creating connections, forums and visibility for young people enabling transitions, communication and the planning of prevention, support, and treatment. But at the same time, the above quote problematizes that we might be putting too much trust in collaboration as the panacea to solve all the problems within the youth mental health care system.

Another critical stance to collaboration was articulated by the School Health Manager in Region 3.

Let’s say that as a young person you sit and talk to a counsellor, and then I see that counsellor come down to my junior high school. ‘Why is she…? What is she doing there?’ It can be like this… A little short circuit in… That it can create a concern. But I think that that is… You can talk about it. It’s an obstacle you can talk about, so it’s nothing strange like that. But some… Absolutely. Some teenagers express it clearly¨.

School Health Manager, Region 3

As the above quote expresses, there is a risk that dense collaboration between services could mean that a young person meets one professional in different spaces and with different roles (e.g., as a therapist at one time and a mental health promoter at another). This situation may not be comfortable for some young people.

## Discussion

The 42 interviews with health professionals working in different parts of the youth mental health system in Sweden, demonstrate the supreme importance that professionals attribute to cross-boundary collaboration. Collaboration was seen as a key strategy to share and negotiate knowledge and responsibilities between actors. Yet, participants were not able to prioritize collaboration and they also saw that close collaboration can have negative implications in relation to young people’s autonomy and privacy.

Cross-boundary collaboration was linked to diverse purposes and meanings and was, for example, used as a strategy to facilitate young people’s navigation across the health system and to reach groups that health services had previously failed to reach. Another purpose of collaboration was to illustrate and compensate the shortcomings of an under-resourced youth mental health system. Collaboration was occurring in multiple layers unfolding in various activities (e.g., arranging and participating in large and small meetings, writing referrals), relationships (e.g., formal, or informal, dense, or shallow collaborative relationships) and target levels (e.g., at individual, group, and organizational levels).

Finally, health professionals shared their insights on the challenges and barriers of collaboration, i.e., conflicting views on youth mental health, the lack of managerial agreements concerning collaboration, and the heavy workloads of professionals operating in an under-resourced youth mental health system. They also saw the possibilities of developing collaboration in two directions. The first was by making formal agreements about collaboration on the managerial level, and the second was by relinquishing organizational boundaries and roles involving collaborative activities, thereby cutting across the isolating ‘drainpipes’.

## Collaborators as boundary spanners

Several manifestations of collaboration in the youth mental health system can be linked to the concept of boundary spanning [43]. Boundary spanning has been suggested as an important mechanism when enhancing collaborative care for mental health [44]. This concept refers to the process of facilitating connections and interactions between individuals, groups or organizations that are separated by a gap of some sort. Boundary spanning can be physical (e.g., geographic location), cognitive (e.g., conceptualizing youth mental health differently) or cultural (e.g., differences between disciplines or professions). The gaps can also result from a lack of trust between the organizations [43]. All types of gaps were identified in the interviews.

In a recent study of the Swedish youth mental health system, focusing on the role of national level stakeholders and the implementation of youth mental health policys, the firm divisions, roles and responsibilities between actors within the youth mental health in Sweden, contributed to isolating gaps between collaborators [26].

This study shows how professionals sometimes act as boundary spanners by filling gaps within the youth mental health system. Despite recognizing the positive aspects of boundary spanning here, there are also potential disadvantages e.g., prompting ambiguity, role conflicts [45] and work stress [44]. These were also seen by the participants as challenges and problems.

### The essential drivers of collaboration: leadership, consequential incentives, and interdependence

The collaborative governance regime is initiated by essential drivers that serve as the impetus for collaboration [37]. The construct of drivers is useful to understand the value and importance that participants attributed to certain features that surround collaborative processes in the youth mental health system. According to the IFCG “one or more drivers of leadership, consequential incentives, interdependence, or uncertainty are necessary for a collaborative governance regime to begin. The more drivers present and recognized by participants, the more likely a collaborative governance regime will be initiated.” [37, p10]. *Leadership* refers to the decisions, support, and resources that leaders engender in collaborative efforts. In our study we found that the leaders had implicit and explicit wishes and expectations on cross-boundary collaboration, which served as the motivation and drivers of collaboration activities. To some extent leaders also facilitated and created the prerequisites, but in many instances, they missed opportunities to create the necessary preconditions for cross-boundary collaboration.

The importance of the leader’s role in cross-boundary collaboration has been shown in several studies [46, 47]. In particular, relational and reflexive abilities, “the capacity to develop trusting relationships with individuals and groups across diverse identities and professional boundaries” [47, p.89], have been highlighted as important leader capabilities for facilitating cross-boundary collaboration. Further, cross-boundary leaders need to be able to prioritize and manage organizational processes that acknowledge and respect different perspectives [48]. The findings from our study indicate that leaders balance different values, e.g., work environments for their staff, in realizing the own organizational assignment and goals. Collaboration, even though expressed as important, was often not prioritized. In some cases, mistrust and heavy workloads meant that individuals tended to guard organizational boundaries rather than cross them.

Another type of driver is *consequential incentives.* This refers to the role of internal or external incentives for collaboration, e.g., problems, resource needs, interests, threats, or crises [37]. This is a strong driver that was evident from the interviews and it is relevant for understanding the many meanings and purposes of collaboration that are manifested in the youth mental health system. The problems, challenges, and threats in the youth mental health system (e.g., complex health needs, gaps in service access, and a shortage of resources) was a clear incentive for engagement in cross-collaborative processes. The concept that cross-boundary collaboration could contribute to solving problems such as poor continuity in youth services, and reaching groups that otherwise would not be reached, was a strong feature noted by the participants. It was also clear that cross-boundary collaboration entailed opportunities for learning, sharing and growing possibilities to ‘do more’ together.

In contradiction, the problems and threats also constituted a barrier for collaboration. It appears as if problems and challenges up to a certain level can motivate collaboration. Yet when organizations are put under heavy stress for long periods of time, the incentives turn into barriers. In a study of barriers to collaboration in the mental health setting Kaas et al. [49] found that when families run out of hope, collaborations face major barriers. This is also a possible mechanism relevant to understanding health professionals’ tendencies to withdraw from collaboration when needs become too large or resources are too limited [49].

The third type of driver is *interdependence* which refers to situations or questions that individuals/organizations could not solve on their own [37]. This driver is an obvious and distinct incentive for the experiences that were reported in our interviews. It is connected to the idea that youth mental health is intrinsically complex and that many different types of resources, perspectives and professional groups are needed to respond to youth mental health needs. What the IFCG fails to capture is that often interdependence is not achieved; for example in our study not all key actors in youth mental health ‘came to the table’ and interdependence, in that sense, lacks relevance or is not a strong enough incentive. Poor supportive structures within an organization can, despite the best intentions, make it impossible to achieve collaboration [50].

This also connects to the final driver of *uncertainty.* Uncertainty in the case of youth mental health relates to unclear roles and responsibilities. The overall imbalance between needs and resources in the system, and the uncertainty on how to best strengthen youth mental health, also constitutes a major uncertainty. This uncertainty is a driver for the collaboration, but again the uncertainty also works both ways. Too much uncertainty about the commitment by youth mental health actors leads to a lack of trust and hinders collaboration.

### Collaboration dynamics: principled engagement and shared motivation

Collaboration dynamics is a set of processes that produce and cultivate iterative cognitions, actions and interactions within the collaborative governance regime. Collaborative dynamics grows through three interacting components - *principled engagement, shared motivation*, and *capacity for joint action* [37].

Collaboration holds an elevated and very special position at all levels in the youth mental health system. Our results show convincingly that collaboration is a cornerstone in providing better, continuous support for all young people in relation to mental health and that the realization of collaboration occurs through joint activities and relationships at different levels within the organization. This also involves activities of discovery and definition of joint interests, problem formulation and mutual expectations. Collaboration facilitates service access for young people and establishes opportunities for the negotiation of power and the sharing of knowledge and responsibilities. The idea of collectively solving difficulties was robustly manifested among the participants and has roots in the political discourses of sharing resources and workloads within the public funded health system [51]. This part of our results is clearly linked to drivers and incentives, as mentioned above, but it also relates to the component *principled engagement.*

The concept of principled engagement suggests that collaboration evolves over time under an iterative social dynamic learning process in which stakeholders develop a shared sense of purpose and also a shared idea on how to realize this purpose [37]. Some of the collaborative processes encountered in our interviews were short (sometimes taking place on one occasion) and informal and did not include the same individuals. It was also stated that it was difficult to get the “right people to the table”, due to high workload and staff turnover. This raises the question: to what extent is collaboration in the youth mental health system hampered by the current instability and high staff turnover in health organizations?

The participants in our study described how formal and overarching agreements can create incentives for functional collaboration but also that personal relationships on the individual level could play an important role. One specific type of collaboration mentioned in our interviews are the CIP-meetings that is regulated by Swedish law and thereby “forcing” relevant actors to the table. A study of children and parents’ experiences of participating in CIP, highlights the importance of relationships, personal support, and the need for CIP-meetings to be personally tailored to realize its full potential [52]. As these studies illustrate, formal and informal incentives serves a complex influence over the collaborative processes.

The second component of the collaborative dynamics is shared motivation which relates to the self-reinforcing cycle of mutual trust, understanding, internal legitimacy and commitment [36]. The aspect of trust among members holds a central position for shared motivation. Trust in turn builds upon the understanding, legitimacy, and commitment that participants experience within the collaboration. In the studied collaboration we had several examples of shared motivation characterized by mutual trust and respect, including respect of other participants’ knowledge, perspectives and boundaries. Yet there are also examples of collaborations where there is mistrust in other participants’ willingness and ability to take part in the collaboration.Collaboration: only a good thing?

Despite the many positive experiences and often high expectations on collaboration, some participants raised concerns that collaboration, in some instances, could imply a shift from the youth-centered approach to a more professional focus. This raises questions about the involvement of youth in collaborations around matters that concerns them. In the study Hedberg et al. [52], children and youth seldom or never participated in the collaborative meetings. The reasons for youth’s non-participation were that parents and the youth themselves anticipated the CIP-meeting to be stressful. Further, parents had been advised by professionals that the children and youths should not participate CIP-meeting but rather be informed afterwards [52]. The findings from both these studies raise questions on if and how children and youth are given possibility to participate in collaborative meetings that involve them.

While Emmerson’s IFCG [37] helps us understand several components of collaborative processes, it fails to problematize the representation of collaboration as essentially and undisputable good and as ‘THE’ way to address the multiple problems of the youth mental health system. The representation of collaboration as the only way to address complex or difficult problems, is present in the literature along with strong arguments for collaborative networks [53]. However, we want to nuance this common view that collaboration is the panacea for addressing complex problems. We agree that collaboration can be an important strategy for addressing such problems, but it the view that collaboration is a desirable end in itself and a way of solving problems is debatable. As our results show, fruitful collaboration also requires smooth functioning within organizations, adequate resources and this is not always the case in youth mental health care.

There has also been some questioning of the viewpoint that collaboration is a solution in Swedish social policies [54]. A possible downside of putting so much faith and belief in collaboration as the solution to a complex health system problem, is that this can close off paths to solutions. Emphasis on collaboration may have a functional purpose because it spreads out responsibility to several or all actors in the health system [55]. However, this withdraws attention from the structural problems which take low priority and can result in an under-resourced mental health system with a shortage of staff, quality and competence [5, 6, 56, 57].

### Strengths and limitations

The 42 participants (key informants) came from a variety of professions, locations, and services/organizations. This enriched and widened the diversity of experiences and perspectives in the data. It can also be considered too large material for ensuring a comprehensive qualitative analysis. However, the analysis focused on the parts of the interviews addressing aspects of collaboration, limiting the scope of the analysis, and allowing for a deeper exploration.

Since this study is part of a larger research project with a focus on YCs, the number of participants from these services is larger, compared to the number of participants from other services, which may have influenced the results. However, collaboration emerged as an aim worth exploring during the data collection. We conducted the analysis iteratively with the data collection, which allowed us to refine the themes following an emergent design - preliminary themes were further explored and deepened in subsequent interviews.

In this study we aimed to investigate how collaboration is understood and practiced by professionals in the youth mental health system and we operationalized this on regional and local levels of the system. We have not explored the national actors perspective although national level actors might serve an important influence over the regional and local levels through mechanisms of national governance, for example policy and legislation.

Conceptual frameworks can be used either directly or indirectly in qualitative research. In this study we began the analysis inductively; the IFCG was introduced after the preliminary themes were developed. We selected those parts of the IFCG that were more applicable for elucidating the results. While the IFCG is often employed in a more directed deductive way, the focus here was on using it as a guide, thereby allowing boundary spanners and other relevant concepts to emerge.

## Conclusion

Collaboration between the services and organizations that work and/or advocate for youth mental health in Sweden serves the purposes of: (i) comprehensively addressing a complex problem; (ii) facilitating the navigation within a complicated network of services; (iii) negotiating and sharing responsibilities and knowledge within and between youth mental health services; (iv) enhancing accessibility and reaching out to young people who are more in need and have less service access, and (v) ensuring that gaps existing within the under resourced youth mental health system are visible and addressed.

These collaborations are realized through diverse activities that address prevention, promotion, and treatment, with a focus on the latter by, for example ensuring referrals to specialized services. Collaboration takes place through a network of relationships that can be formal or dense, to varying extents. This takes place at the level of the individual user, between different professionals and/or at the managerial or system level.

Collaboration faces several challenges, including: (i) the difficulty in harmonizing services with diverse perspectives on youth, mental health, and collaboration; (ii) the lack of agreements at managerial level that makes collaboration highly dependent on individuals’ commitment and hinders sustainability, and (iii) overload in the youth mental health system making it difficult to prioritize collaborating activities and meetings. When it works at its best, collaboration is grounded on overarching formal agreements and committed professionals who: (i) trust each other; (ii) have knowledge and appreciation for each other’s diverse tasks and expertise; (iii) rely on personal closeness but do not depend on specific persons for functioning, and (iv) have time and resources and ways of collaborating. Finally, even if collaboration is represented as being worthy and good, there are some instances when too much or close collaboration can hinder youth privacy and autonomy, especially when the focus and decision-making power is centred around the professionals rather than young people who should be at the centre.

Collaboration has been suggested as a strategy to battle gaps in quality and accessibility that are evident in parts of the Swedish youth mental health system. Drawing on the results of our study we conclude that collaboration alone will not fully compensate for an under-resourced youth mental health system.

### Electronic supplementary material

Below is the link to the electronic supplementary material.

Supplementary Material 1

Supplementary Material 2

## Data Availability

The dataset used for this study is not publicly available since it contains sensitive information, but it is available from the corresponding author on reasonable request.

## Abbreviations

IFCG: Integrative Framework for Collaborative Governance
