# Supplementary material for: Exploring cross-boundary collaboration for youth mental health in Sweden – a qualitative study using the integrative framework for collaborative governance
Source: BMC Health Serv Res. 2024 Mar 11;24:322. doi: 10.1186/s12913-024-10757-y (PMC10929090; doi:10.1186/s12913-024-10757-y)
Supplement: Supplementary file 1 — Supplementary Material 1 [file 12913_2024_10757_MOESM1_ESM.docx]

## Governance of the Swedish youth mental health system and the three regions in Sweden included in the study

The responsibility for providing health services in the Swedish healthcare system is shared between the national government, the 21 regions and the 290 municipalities. In addition, government agencies, such as the National board of health and welfare, the Public health agency and the Swedish Agency for Youth and Civil Society, support both the government and the health care providers by offering statistics and knowledge. Legislation, such as the Health care act regulates health care practice. The Swedish Healthcare Act commits to provide ‘good health and care on equal terms for the entire population’.

The health care act also states that collaboration between actors within the youth mental health system in some situations are mandatory by law, i.e. Coordinated Individual Plan (CIP). When an individual needs services from several actors in the welfare system, e.g. from health care and the social services. The legislation describes what a CIP must contain, and that the individual has the right to be involved in the work.

Data was collected in three regions in Sweden. Region 1 (figure S1) is situated in the northern regions of Sweden and significantly smaller in terms of population (app. 250 000 habitants as of 2021) with the consequently smaller budget and fewer youth mental health actors. Participants in this region included professionals working in youth clinics (YC), school health, social services, primary health care centres, child and adolescent psychiatry, adult psychiatry. In addition to professionals, we also included leaders of two civic organization engaging young people were included in the case in addition – one local theatre and a youth café. Organizations within dotted lines are former or planned associations.

Region 2 (figure S2) is located in the south of Sweden, with a population of approximately 1 700 000 habitants (as of year 2021). Participants in this region consisted of professionals working in YCs, school health, specialized youth mental health services, leisure organizations, youth health promotion organization, gender agency and trauma agency. In addition, we included the leader of a local youth club working with LGBTQ young people and a youth psychotherapy organization.

Region 3 (figure S3) is located in the middle of Sweden, with a population of approximately 300 000 inhabitants. Participants in this region included professionals working in YCs, in school health, in specialized youth mental health services and leisure organizations.

To be able to describe the purposes, meanings, and challenges of collaboration, the “collaborative landscapes” of youth mental health in Sweden, will be introduced briefly (figure S1, S2 and 3). YC holds a central position, and around them health care, social care and civic organizations are operating.

The collaborative landscapes for region 1 and 2 share several features. The YCs in both region 1 and 2, have two internal teams – a mental health team and a sexual health team, they work independently but also very connected and in collaboration. Collaboration, in both regions also involves the community level with actors as schools, youth clubs and social services, a health care level divided in primary health care with health care centers and a specialized level including child- and adolescent psychiatry and adult psychiatry. Finally, both regions included civic society, represented by for example theatre groups. Thera are also some differences between the collaborative landscape in the two regions. Region 1 involves fewer actors and informants also refers to organizations that not yet, or no longer exist.


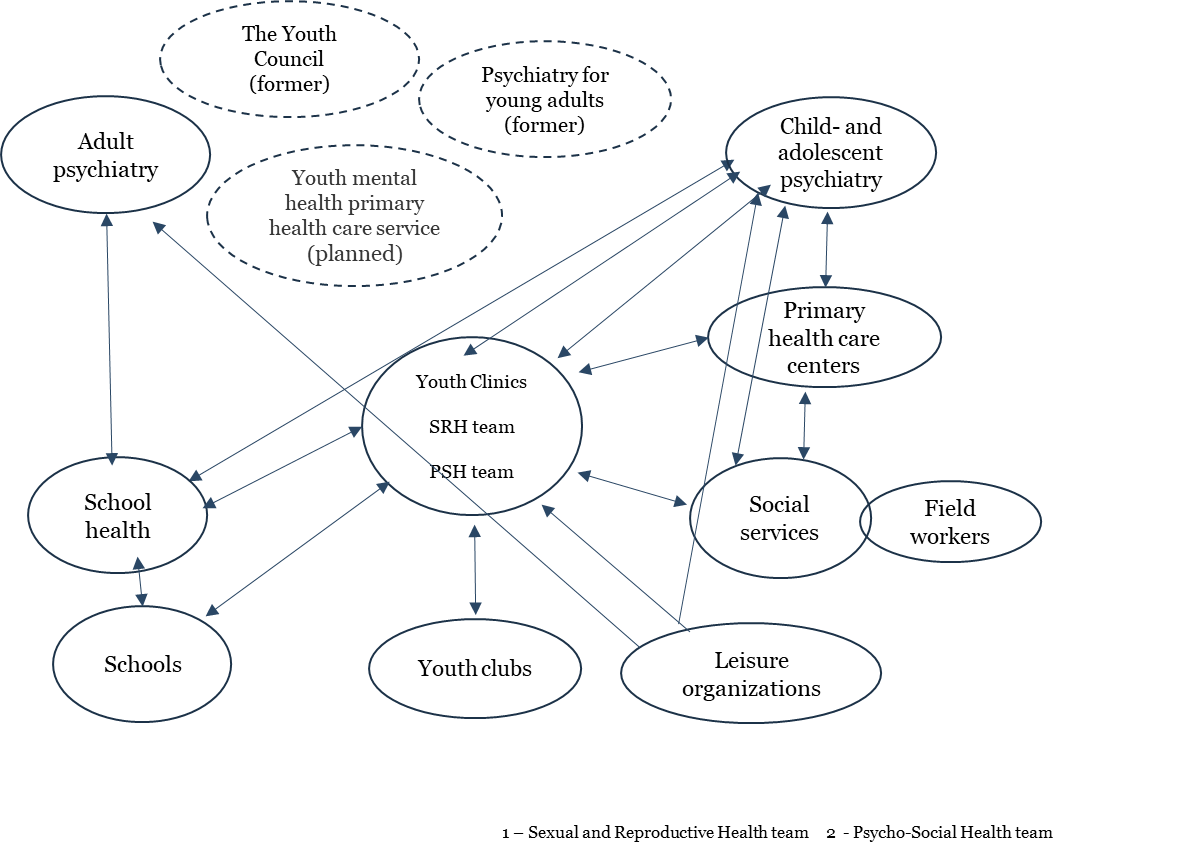


Figure S1. Collaborative landscape, Region 1


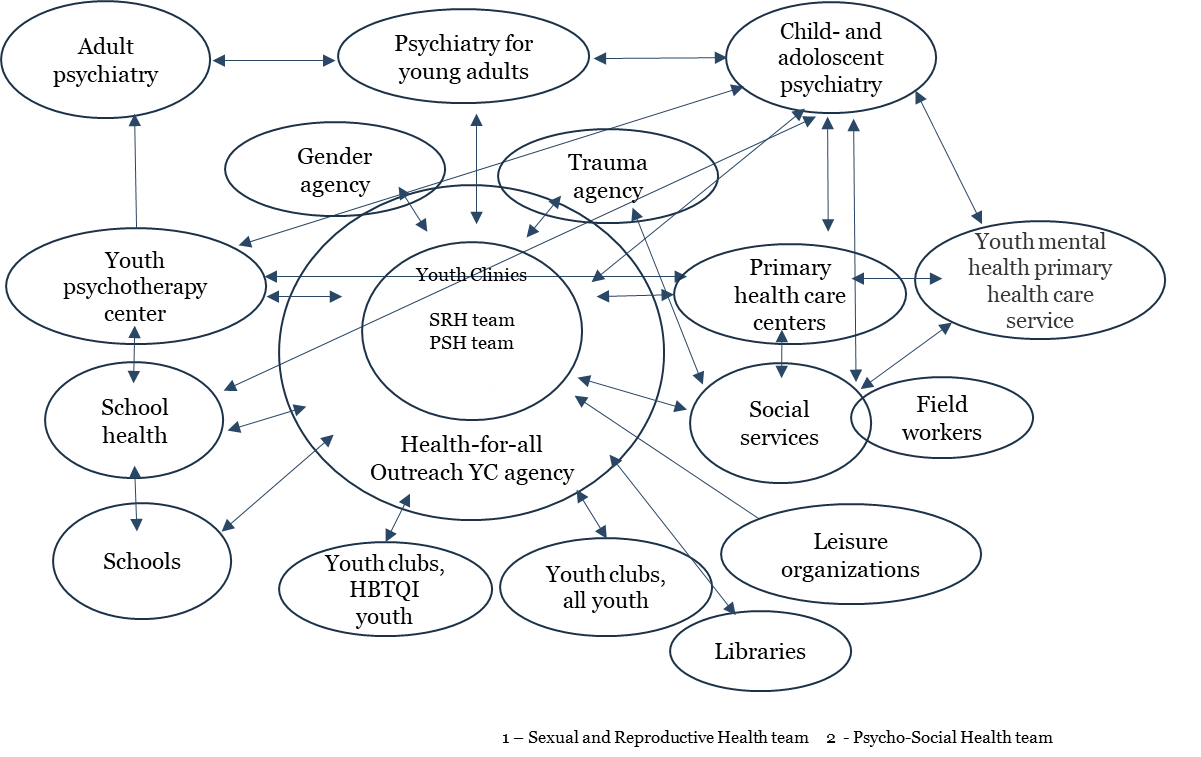


Child- and adolescent psychiatry

Figure S2. Collaborative landscape, Region 2


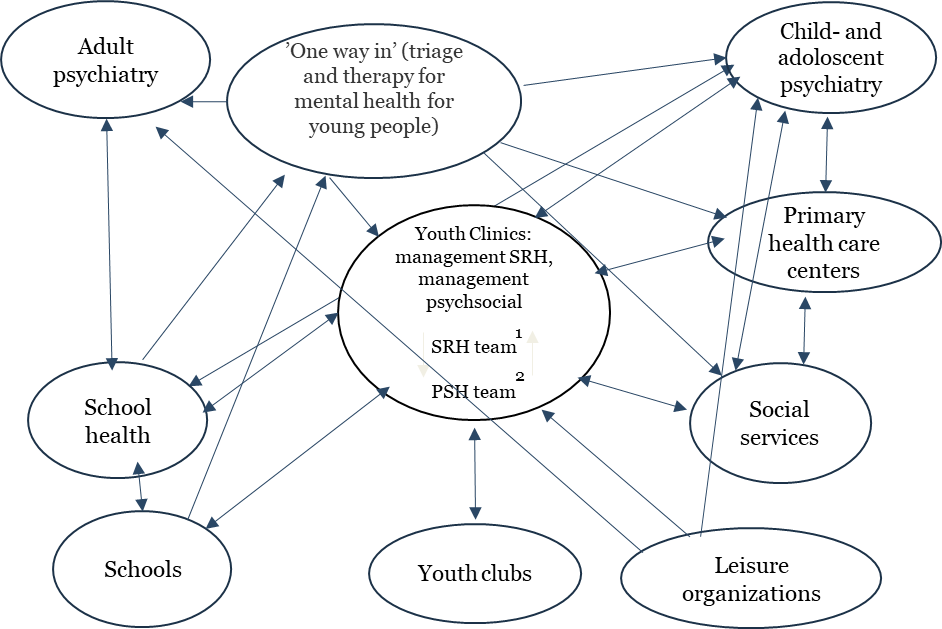


Child- and adolescent psychiatry

Figure S3. Collaborative landscape, Region 3
