# Supplementary material for: Exploring cross-boundary collaboration for youth mental health in Sweden – a qualitative study using the integrative framework for collaborative governance
Source: BMC Health Serv Res. 2024 Mar 11;24:322. doi: 10.1186/s12913-024-10757-y (PMC10929090; doi:10.1186/s12913-024-10757-y)
Supplement: Supplementary file 2 — Supplementary Material 2 [file 12913_2024_10757_MOESM2_ESM.docx]

## Semi-structured interview guide – Professionals working in Youth Clinics

1. Would you like to start by telling us about your professional role in the Youth Clinic? (Follow up: How long has the participant worked in the YC? Additional roles?)
2. What type of questions/concerns young people bring to you? (Possible follow up: Do you meet young people who have questions concerning their mental (ill) health?)
3. Describe the Youth Clinics responsibility and role for young people's mental illness/health? (Possible follow up: The assignment, the role, working methods and methods, scope)
4. What is the purpose of collaboration in your work on youth mental health? (Possible follow up: The role of collaboration on youth mental health? Benefits, downsides, short- and long term?)
5. When collaboration around youth mental health occurs, how is it practiced, carried-out? (Possible follow up: Who do you collaborate with? At what stage in the process? On what topics? How is collaboration carried out in practice?)
6. When do collaboration on youth collaboration work well? (Possible follow up: What do you define as ‘collaboration working well’ in this context? What are the signs of collaboration working well? What conditions do participants connect to collaboration working well?)
7. Does it happen that collaboration fail or that it ‘works less good?’ (Possible follow up: What do you define as ‘collaboration fail/ not working well’ in this context? What are the signs of collaboration failing/not working well? What conditions do participants connect to collaboration fail?
8. (If not responded to earlier) What are the barriers of collaboration on youth mental health?
9. Any additional things or other aspects you would like to add?

Thank you!
